# Giant solitary fibrous tumour of the pleura. Case report and review of the literature

**DOI:** 10.2478/raon-2014-0036

**Published:** 2015-11-27

**Authors:** Anton Crnjac, Bojan Veingerl, Damjan Vidovic, Rajko Kavalar, Aljaz Hojski

**Affiliations:** 1 Department of Thoracic Surgery, Division of Surgery, University Medical Centre Maribor, Slovenia; 2 Department of Pathology, University Medical Centre Maribor, Slovenia

**Keywords:** solitary fibrous tumour of the pleura, expansive growth, mediastinum shift, surgical treatment

## Abstract

**Background:**

Solitary fibrous tumours of the pleura (SFTP) are rare tumours. They are mostly benign. Only around 12% of them are malign ant. In the initial stage they are mostly asymptomatic and by growing they cause chest pain, irritating cough and dyspnoea on account of the pressure created on the surrounding structures. Rare giant tumours have compression symptoms on the mediastinal structures. The condition requires tiered diagnostic radiology. Preoperative biopsy is not successful in most cases. The therapy of choice is radical surgical tumour removal. Malignant or non-radically removed benign solitary fibrous tumours of the pleura additionally require neoadjuvant therapy.

**Case report:**

A 68-year old patient was hospitalized for giant solitary fibrous tumour of the pleura in the right pleural cavity. With its expansive growth the tumour caused the shift of the mediastinum by compressing the lower vena cava, right cardiac auricle as well as the intermediate and lower lobe bronchus. Due to cardiac inflow obstruction and right lung collapse, the patient’s life was endangered with signs of cardio-respiratory failure. After preoperative diagnostic radiology, the tumour was surgically removed. Postoperatively, the patient’s condition improved. No disease recurrence was diagnosed after a year.

**Conclusions:**

Giant solitary fibrous tumour of the pleura may cause serious and life-threatening conditions by causing compression of the pleural cavity with its expansive growth. Early diagnosis of the condition enables less aggressive as well as video-assisted thoracic surgery in patients with significantly better state of health. Large tumour surgeries in cardio-respiratory affected patients are highly risk-associated procedures.

## Introduction

Solitary fibrous tumours of the pleura (SFTP) are rare mesenchymal tumours representing less than 5% of all tumours of the pleura.^1^ Around 800 cases of such tumour types have been mentioned in global literature up to date.^2^,^3^ In 1870 Wagner was the first to describe a localized primary tumour of the pleura^4^, Klemperer and Rabin first classified them in 1931 into a diffuse and localized form and set a hypothesis that localized mesothelium, covering the intact layer of mesothelial cells, stems from structures under the mesothelial layer.^5^ With the introduction of electronic microscopic and immunohistochemical examinations it was finally confirmed that SFTP grow from deeper-lying mesenchymal structures of the thoracic wall.

Historically, SFTP are thought to derive from subpleural mesenchymal cells with fibroblasts or myofibroblast differentation.^6^ However, negative staining for smooth muscle markers and diffuse positivity for CD34 led van de Rijn *et al.* to propose an origin of this tumour from ubiquitous dispersed dendritic interstitial cels.^7^ Recent ultrastructural observations have highlighted that SFTP may originate from peculiar perivascular multipotent mesenchymal elements displaying features akin to pericytes and submesothelial fibroblasts.^8^ To differentiate these tumours from other soft tissue tumours, immunohistochemical examinations are required. Tumours with similar histological characteristics are described by certain authors also in extrathoracic organs, such as liver, peritoneum, meninges, orbits, thyroid gland, salivary gland, breast.^9^–^12^

SFTP affect male and female patients equally; however, they may develop in all age periods (5 to 87 years) with the highest incidence between 60 and 70 years of age.^13^,^14^ Generally, there is no evidence of correlation with the genetic predisposition for the tumour, and in contrast with mesothelioma^15^, there is no relationship to the exposure to asbestos, tobacco or any other environmental agents.^16^

Most SFTP are benign, but may alter in malignancy with age. They usually develop in lower part of pleural cavity; from the visceral pleura in around 80%.^16^ Individual tumours grow over 10 cm; however, only individual cases of giant tumours measuring over 20 cm are described.^1^ Cytological and histological diagnosis as well as differential diagnosis by defining the benign or malignant biological potential is difficult to perform with the bioptic material acquired by means of fine-needle aspiration biopsy or with large-core needle biopsy. The final diagnosis is usually made after the tumour has been removed. England *et al.* set the basis for differentiating benign from malignant SFTP (Table 1).^13^ Based on the histological and morphological characteristics of SFTP, De Perrot *et al.* classified tumours into five stages and thus enabled easier planning of therapy and expected outcome of treatment (Table 2).^2^ Most minor SFTP are asymptomatic. They are usually discovered incidentally during chest X-ray examination.^17^ By growing and pressuring the surrounding structures they become symptomatic. The most common clinical signs are coughing, dyspnoea, and chest pain, especially in tumours growing from parietal pleura. Rarely are they manifested with the signs of haemoptysis, obstructive pneumonitis or atelectasis.^18^ In larger tumours, digital clubbing and hypertrophic pulmonary osteoarthropathy (Pierre Marie Bamberg syndrome)^19^ may be present or signs of refractory hypoglycaemia on account of insulin-like growth hormone release (Doege Potter syndrome).^20^ Extremely large tumours cause a variety of clinical signs of pressure on the mediastinum or mediastinal shift.

Diagnostic radiology plays a very important role in discovering SFTP. Chest radiographs of patients demonstrate a well-defined, solitary nodule or mass, which may appear at the lung periphery and typically abuts the pleural surface or is located within a fissure.^21^ Pedunculated tumours may show mobility within the pleural space.^14^ Computer tomography (CT) of the chest shows a homogenous, well-defined and lobulated soft tissue mass.^20^ In cases with suspected infiltrative tumour growth into the mediastinal structures, like in others pleural tumours, magnetic resonance imaging (MRI) is required.^22^,^23^ Because such tumours are well-circulated, it seem sensible to perform angiography.^24^ Lately, it has been recommended to perform a PET-CT scan, especially when suspecting malignant SFTP or to confirm the presence of potential metastases.^25^

Radical surgical resection is the optimal way of treating patients with SFTP. Aggressive surgery is recommended due to the high probability of their recurrence.^26^ The safety margin of healthy tissue after resection should be 1–2 cm wide. Wedge resection of the lung and limited pleurectomy may suffice in peripheral tumours. For sessile tumours it is necessary to perform a lobectomy or pneumonectomy as well as extensive pleurectomy, sometimes even partial resection of the chest wall.^27^ Smaller, especially pedunculated tumours, can also be radically removed with minimally invasive thoracoscopic surgical procedure (VATS), which is routinely use in different thoracic pathologies.^28^ In cases of larger SFTP, the continuation of surgical treatment with adjuvant chemotherapy is indicated.^27^,^29^ Park *et al*. have found that the combination of temozolomide and bevacizumab had high rates of overall response and long term disease control.^30^ In their study, patients received temozolomide 150 mg/m^2^ orally on days 1–7 and days 15–21 and bevacizumab 5 mg/kg intravenously on day 8 and day 22 on a 28-day cycle.^30^ The role of brachytherapy and photodynamic therapy, a method in treating diffuse mesotheliomas, has not been sufficiently studied.^31^

## Case report

68-year old female patient was admitted to the Department of Lung Disease with signs of severe cardio-respiratory failure. One month prior to being admitted, the patient’s breathing was getting heavier, she was tired, weak, had poor appetite and increasing pain in the right hemithorax. She also had arterial hypertension and atrial fibrillation. She has never smoked.

Written informed consent of patient was obtained for the treatments and for the scientific use of clinical data, according to Declaration of Helsinki and Slovenian law requirements.

At examination the patient was cyanotic, tachypnoic with the breathing frequency 22/min. With the administration of oxygen by a nasal catheter a peripheral capillary oxygen saturation (SpO2) was 91%, the patient had signs of heart failure with atrial fibrillation. Breathing was weakened and audible only apically on the right side. Pulmonary function test showed a significant decrease in values of the forced vital capacity (FVC, 42% of the norm), the forced expiratory volume in 1 second (FEV1, 35% of the norm), and the FEV1/FVC ratio or Tiffeneau index (TI, 68% of the norm). Gas analysis of arterial blood showed signs of chronic hypercapnic respiratory failure.

**FIGURE 1.**
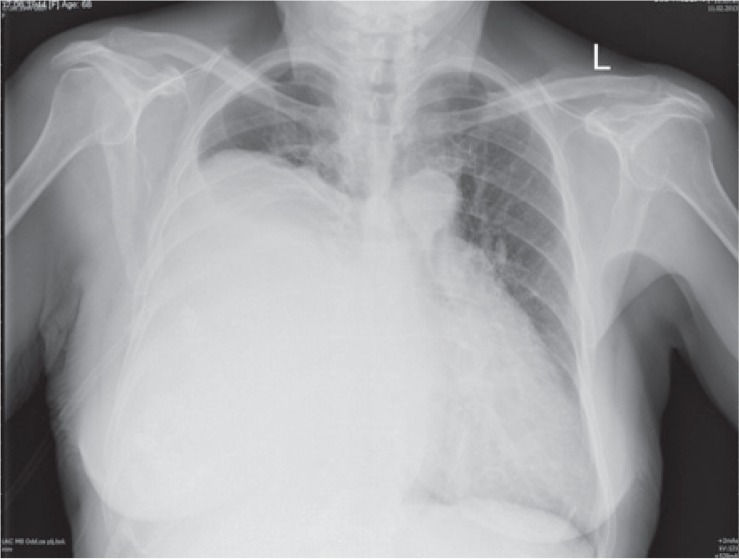
Chest X-ray - a large tumorous process of the right hemithorax with mediastinal shift to the left.

**FIGURE 2.**
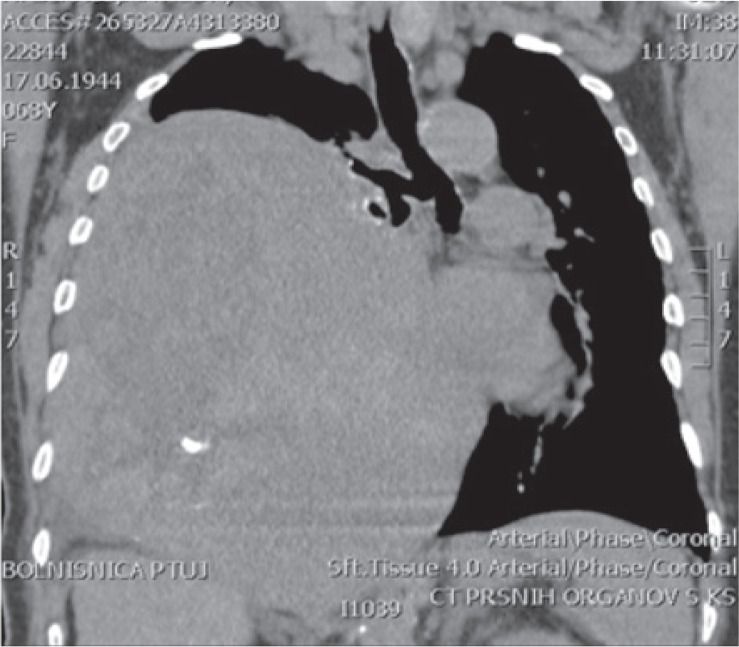
Chest CT scan - non-homogeneous, extensive tumour of the right chest side.

**FIGURE 3.**
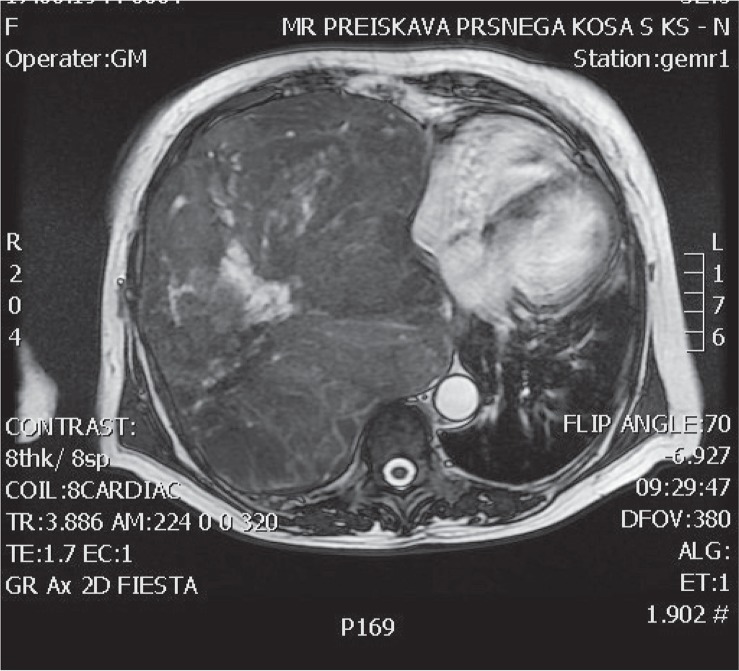
Same tumour displayed using MRI - no signs of overgrowth in the structure of the mediastinum.

Chest X-ray showed a large tumour mass in the right part of the thorax with mediastinal shift to the left (Figure 1). CT scan of the chest showed an extensive expansive process, larger than 20 cm. The tumour was heterogeneous, lobulated and practically extended over the entire right pleural cavity and shifted mediastinal structures to the left (Figure 2). An MRI examination did not confirm tumour infiltration of the surrounding mediastinal structures (Figure 3).

Bronchoscopy showed a visibly compressed trachea from the right side, shift of the carina and the main right bronchus into the left and closed bronchus for the right lower lung lobe. Transbronchial tumour biopsy was negative; the material collected by means of transthoracic needle biopsy did not suffice for histological tumour confirmation.

**FIGURE 4.**
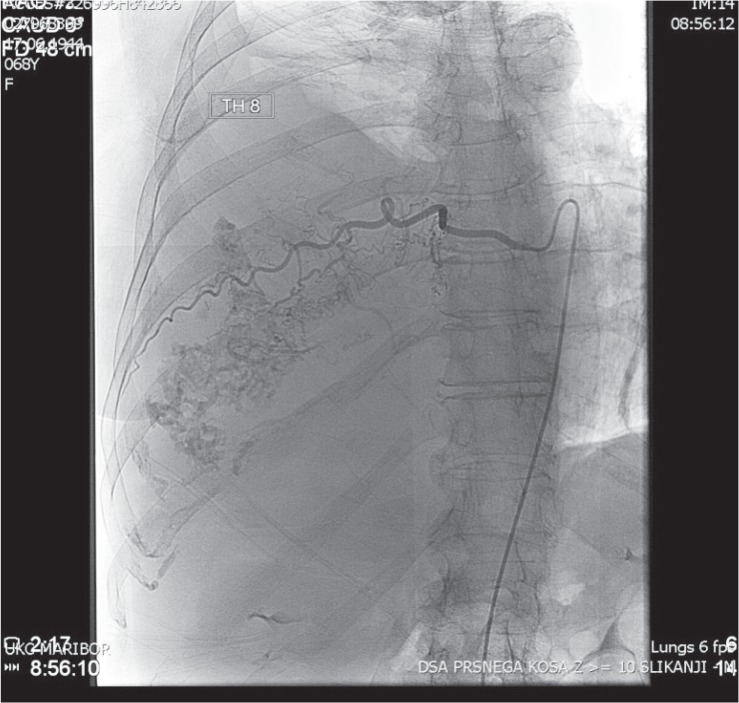
The angiography - perfusion of the tumour from the intercostal arteries and right inferior phrenic artery.

For purposes of further diagnosis and surgical treatment the patient was transferred to the Department of Thoracic Surgery. In the preoperative phase we performed an ultrasound (US) of the heart and angiography of the tumour. Heart US showed a compressed right atrium and lower vena cava as well as increased pressure in the right atrium and ventricle. Angiography displayed an extensive blood circulation of the tumour from the intercostal arteries 8, 9, 11, and 12 and inferior phrenic arteries from the right side (Figure 4).

**FIGURE 5.**
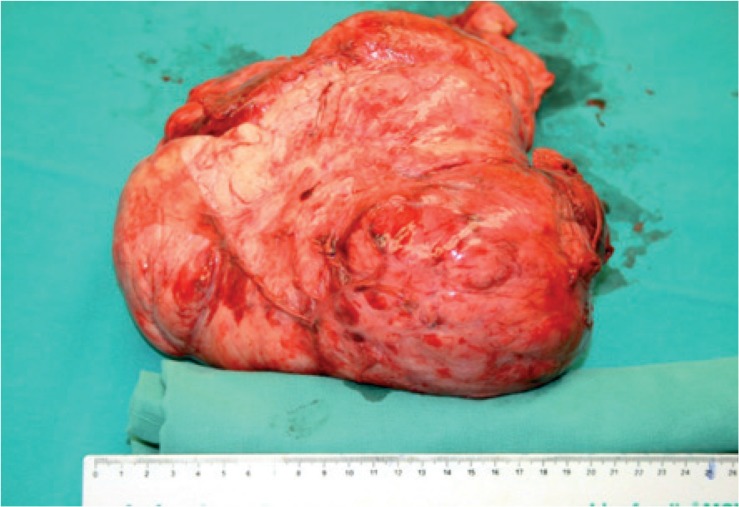
Removed solitary fibrous tumour of the pleura; size 22 × 16 × 15 cm.

Because of the clinical status of the patient with the signs of respiratory failure, cardiac inflow obstruction and the possibility of massive bleeding, all together representing a high-risk surgery, a detailed plan was designed. A wide approach for safe and radical tumour removal was enabled with the right thoracosternotomy (hemiclamshell). By continuous ligation of blood vessels nourishing the tumour, the blood loss during surgery was only 1.5 l of blood, which was recycled by a cell-saver. Surgical preparation in the mediastinal area was difficult because of compressed structures and numerous postinflammatory adhesions. A fully removed tumour was sent for pathohistological examination (Figure 5).

Macroscopic examination of the resected specimen showed firm lobulated and bosselated white-grey tumour measuring 25 cm x 16 cm x 13 cm. The surface of the tumour was covered with thin, shiny, smooth capsule. The cut surface was rubbery, vaguely nodular, grey-white, focally glassy and haemorrhagic. On the edges tiny calcifications were present. Macroscopically no necrotic areas were identified.

**FIGURE 6.**
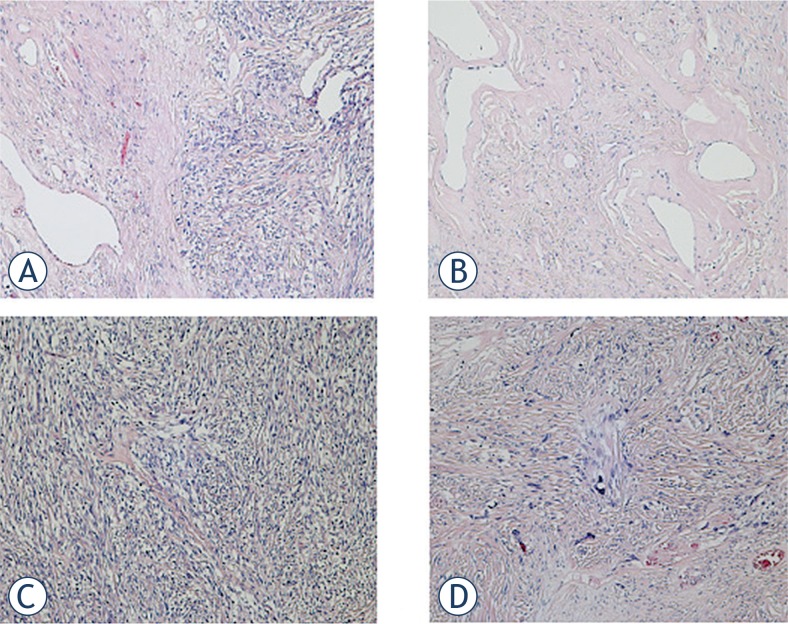
Microscopic features of the tumour. (**A**) Haemagioperycytic pattern in hypercellular and hypocellular sclerotic setting; (**B**) Hyalinised vascular structures; (**C**) Patternless pattern of growth; (**D**) Pleomorphic cells. (A,B,C,D: HE, 100x).

Microscopically the bland appearing tumour cells were arranged in a “patternless pattern” (storiform and fascicular pattern), the hypercellular regions were mixed with hypocellular areas with hyalinised stroma. In some areas stromal myxoid change and degeneration of collagen was present, too. Stroma was highly vascularized with angiofibromatous and haemangiopericytic vascular pattern. The tumour cells were spindle and oval with scant cytoplasm and nuclei with dense chromatin. Focally spindle cells showed wavy nuclei, resembling schwanian cells and also there were some areas with pleomorphic and giant cells population. The nuclei of the pleomorphic cells were larger, hyperchromatic, and different in shape. Very rare mitotic figures (<2/10 high-power fields [HPF]) were present and there was no necrosis (Figure 6).

**FIGURE 7.**
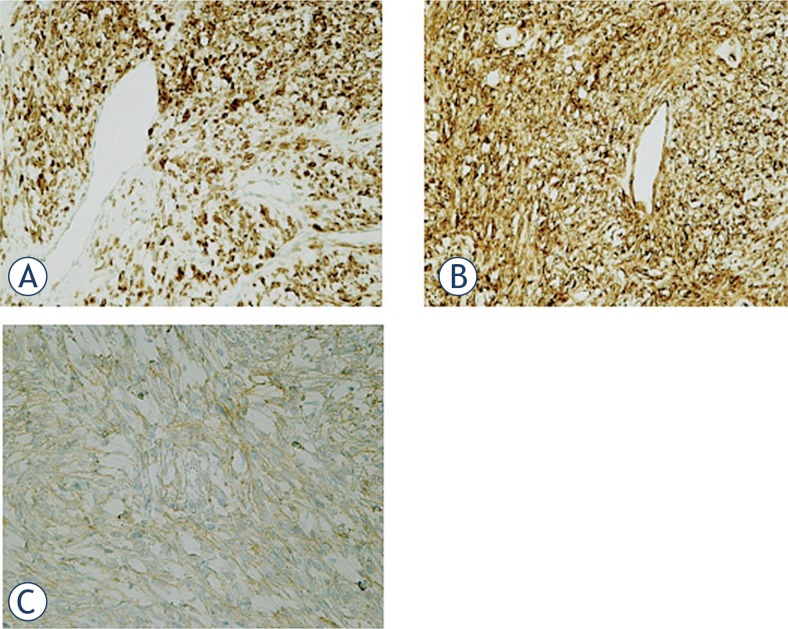
Positive immunoreactivity of tumor cells. (**A**) Bcl2; (**B**) CD34; (**C**) CD99. (A,B,C: 200x).

Immunochistochemically tumour cells were reactive for CD34, CD99 and bcl2 and typically no immunoreactivity was observed with S-100, WT-1, Desmin, CEA, CK AE1/AE3, CK5/6 and calretinin (Figure 7).

According to the morphology and cellular immunophenotype the diagnosis of benign giant pleural SFT was signed out.

The patient was placed for seven days into a room with perioperative intensive care and extubated after two days. Longer intubation was required to ventilate a long time collapsed right intermediate and lower lung lobe. She left the hospital on day 22 after surgery. At the follow-up after a year, no recurrence of the disease was present.

## Discussion

SFTP are a rare pathology of the pleural cavity, which most of the time develop from submesothelial fibroblasts of the visceral pleura and usually in the lower parts of the chest. Prior to the introduction of immunohistochemical examinations and electronic microscopy, they were classified into a large group of mesotheliomas as a localized form of this dangerous, asbestos-related pathology.^16^ Despite the relatively benign disease course, questions remain open in the field of diagnostics, preoperative histological verification and final treatment.

Due to their non-characteristic clinical picture, SFTP are usually diagnosed in the later stages of the development, when causing pressure on the surrounding structures on account of their size. Smaller, accidentally discovered SFTP are relatively easy to remove surgically. A much more significant issue are radical surgical procedures of giant SFTP in patients affected by the pressure of the tumour on the mediastinal structures and lungs. Only a few cases of giant SFTP that cover almost the entire pleural space are described in literature. SFTP that we removed belongs to the largest so far described cases in global literature. Certain authors report of having performed the surgery *via* one or two thoracotomies at two different levels^1^, others via sternotomy.^3^ Our approach *via* right thoracosternotomy (hemiclamshell) provides an optimal view of the pleural cavity and mediastinal structures.

Preoperative diagnostics needs to be systematic to enable the surgeon a precise estimation of the scope of the surgical procedure, tumour operability, blood flow of the tumour and the relation to neighbouring structures.

A CT scan provides valuable data on the exact location of SFTP, its relation to surrounding structures, tumour homogeneity or potential bleeding areas or necrosis, chest wall destruction and the presence of pleural effusion.^21^,^32^,^33^ However, a CT scan cannot differentiate between benign and malignant SFTP cases. Large tumours are more likely to be malignant, with distinct heterogeneous structure, not clearly separated from the surrounding environment and in potential presence of pleural effusion.^21^ A part of the diagnostic preoperative examinations performed in our patient was an MRI examination to exclude tumour infiltration into mediastinal structures and angiography to display the feeding arteries. Similar guidelines are also supported by other authors.^22^,^24^ Pathological arteries usually arise from chest arteries and we found an interesting description of the arterial circulation in SFTP from the celiac and hepatic arteries.^34^

Preoperative histological confirmation of such tumour changes in the chest remains a big problem. The diagnosis of SFTP is rarely reached before surgical excision and pathological examination of the mass.^16^ Sometimes preoperative diagnosis can be made with large-core needle biopsies. The risk of pneumothorax could be minimal with avoiding aerated lung on the introduction of the needle.^35^ Although fine-needle aspiration biopsy may yield characteristic and diagnostic morphological features, it was difficult to reach a histological diagnosis in most studies.^19^ Cutting needle biopsy is probably preferable because of wider tissue sampling.^35^ Thoracoscopic procedure, an effevtive diagnostic and therapeutic method, have also be to considered.^36^ In our case it was not possible to collect suitable material for histological analysis with transthoracic and transbronchial biopsy.

Because in most cases the biological potential of SFTp is not preoperatively histologically confirmed, neoadjuvant therapy is not appropriate. After removing the tumour surgically we opt for adjuvant therapy in malignant or non-radically removed benign SFTP in accordance with the guidelines provided by De Perrot *et al*. (Table 3).^16^ Because the benign SFTP was removed radically, our patient did not receive adjuvant therapy.

It is possible that the tumour will recur, which mainly depends on the histological characteristics of SFTP and radical nature of the surgical procedure. The possibility of benign pedunculated tumours recurring is around 2%, for benign sessile tumours around 8%, malignant pedunculated tumour around 14%, and for malignant sessile tumours around 63%.^31^,^37^ It is necessary to follow the patients, usually every 6 months when control CT scan of the chest is performed. No recurrence was established in our patient after a year.

## Conclusions

SFTP are rare pleural neoplasms, stemming from submesothelial fibroblast cells and are in more than 80% benign. Initially, they are asymptomatic and by growing they create pressure on the surrounding structures of the chest and cause chest pain, coughing, dyspnoea, and by pressuring on the mediastinum they can cause life-threatening signs of mediastinal shift. Tiered diagnostic radiology is very important and provides valuable data in the most appropriate manner of treatment. Preoperative biopsy is usually not successful and the final diagnosis is obtained in most cases only after the surgical removal of the tumour. Radical surgical resection is the method of choice when treating benign and operable SFTP and need to be upgraded in malignant or non-radically removed benign tumours with adjuvant therapy. Extensive surgical resections can be avoided with timely diagnosis of smaller tumours, which can be radically removed with VATS. Regular check-up are required due to possible disease recurrence.

## Figures and Tables

**TABLE 1.** Malignancy criteria for SFTP

**A.** Abundant cellularity with crowding and overlapping of nuclei **B.** High mitotic activity of more than four mitotic figures per 10 high power fields **C.** Pleomorphism with cytonuclear atypia **D.** Large necrotic or haemorrhagic areas **E.** Associated pleural effusion **F.** Atypical location and inversion of adjacent structures

**TABLE 2.** Classification of SFTP into stages

**Stage 0** –peduculated SFTP without signs of malignancy **Stage 1**- sessile or inverted SFTP without signs of malignancy **Stage 2**- pedunculated SFTP with histological signs of malignancy **Stage 3**- sessile or inverted SFTP with histological signs of malignancy **Stage 4**- multiple synchronous metastatic tumours

**TABLE 3.**
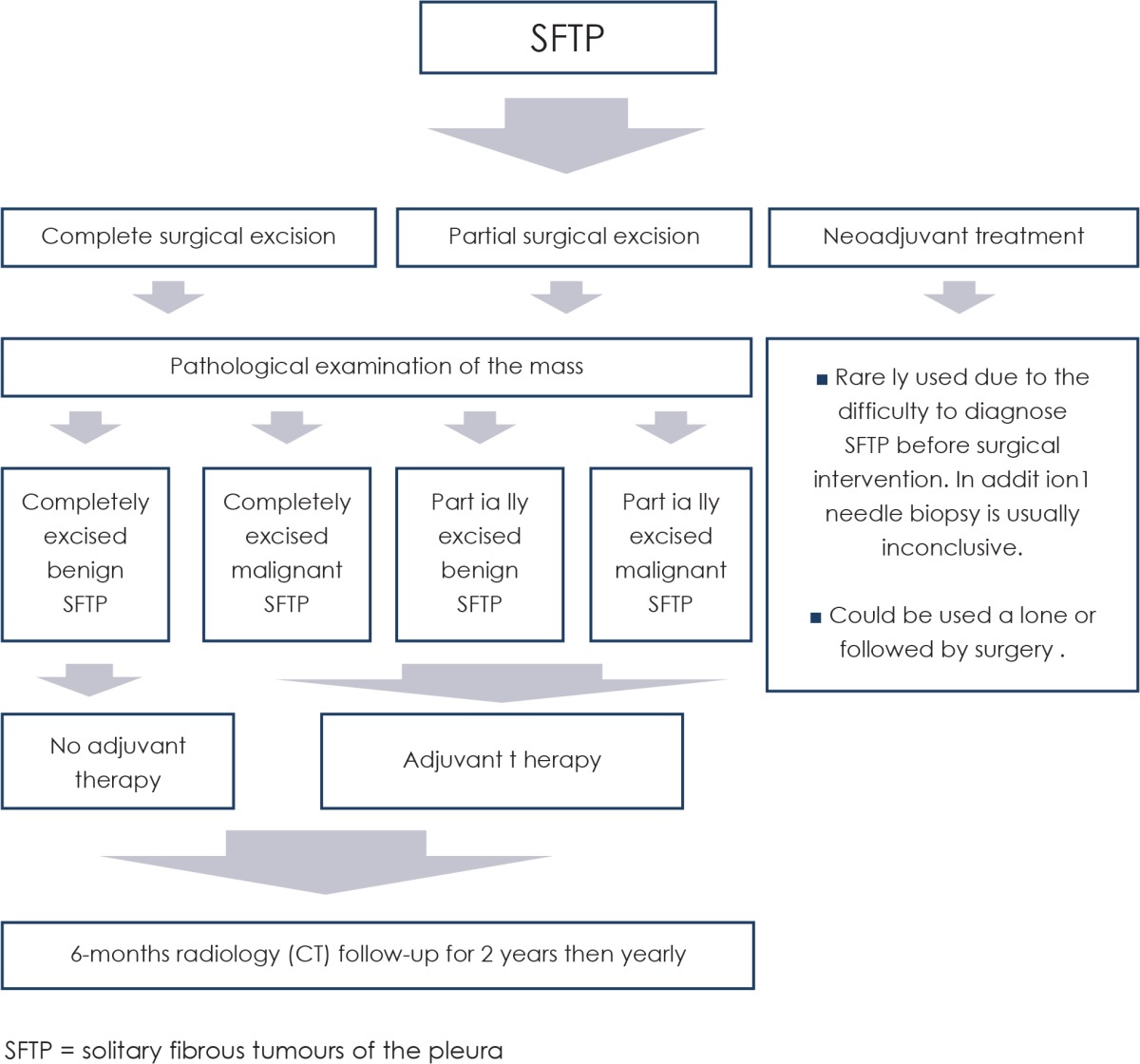
The guidelines provided by De Perrot *et al*.

## References

[1] Furukawa N, Hansky B, Niedermayer J, Gummert J, Renner A (2011). A silent gigantic fibrous tumor of the pleura: case report. J Cardiothorac Surg.

[2] De Perott M, Fischer S, Brundler MA, Sekine Y, Keshavjee S (2002). Solitary fibrous tumors of the pleura. Ann Thorac Surg.

[3] Sung SH, Chang JW, Kim J, Lee KS, Han J, Park S (2005). Solitary fibrous tumors of the plevra. Surgical and cinical course. Ann Thorac Surg.

[4] Wagner E (1870). Das tuberkelähnliche lymphadenom. (Der cytogene oder reticulirte tuberkel). Arch Heilk (Leipzig).

[5] Klemperer P, Rabin CB (1931). Primary neoplasm of the pleura: a report of five cases. Arch Pathol.

[6] Robinson LA (2006). Solitary fibrous tumor of the pleura. Cancer Control.

[7] Van de Rijn M, Lombard CM, Rouse RV (1994). Expression of CD34 by solitary fibrous tumors of the pleura, mediastinum and lung. Am J Surg Pathol.

[8] Ide F, Obara K, Mishima K (2005). Ultrasructural spectrum of solitary fibrous tomor: a unique perivascular, tumor with alternative lines of differentation. Virchows Arch.

[9] Ibrachim NB, Briggs J, Corrin B (1993). Double primary localized fibrous tumor of the pleura and retroperitoneum. Histopathology.

[10] Vaswani K, Guttikonda S, Vitellas KM (2000). Case 1. Localized fibrous tumor of the liver. AJR Am J Roentgenol.

[11] Sandvliet RH, Heysteeg M, Paul MA (2000). A large thoracic mass in a 57-year-old patient: solitary fibrous tumor of the pleura. Chest.

[12] Safneck JR, Alguacil-Garcia A, Dort JC, Philips SM (1993). Solitary fibrous tumor: report of two new locations in the upper respiratory tract. J Laryngol Otol.

[13] England DM, Hochholzer L, McCarthy MJ (1989). Localized benign ena malignant fibrous tumor of pleura: a clinicopathologic rewiev of 223 cases. Am J Pathol.

[14] Dynes MC, White EM, Fry WA, Ghahremany GG (1992). Imaging manifestations of pleural tumors. Radiographics.

[15] Kovac V, Zwitter M, Zagar T (2012). Improved survival after introduction of chemo-therapy for malignant pleural mesothelioma in Slovenia: Population-based survey of 444 patients. Radiol Oncol.

[16] Walid AA (2012). Solitary fibrous tumors of the pleura. Eur J Cardiothorac Surg.

[17] Magdeleinat P, Alifano M, Petino A, Le Rochais JP, Dulmet E, Gaalateau F (2002). Solitary fibrous tumor of pleura: clinical charateristic, surgical treatment and outcome. Eur J Cardiothorac Surg.

[18] Robinson LA (2006). Solitary fibrous tumor of the pleura. Cancer Control.

[19] Rena O, Filosso PL, Papalia E, Molinatti M, Di Marzio P, Maggi G (2001). Solitary fibrous tumor of the pleura: surgical treatment. Eur J Cardiothorac Surg.

[20] Chamberlain MH, Taggart DP (2000). Solitary fibrous tumor associated with hypoglicemia: an example of the Doege-Potter syndrome. J Thorac Cardiovasc Surg.

[21] Dedrick CG, McLoud TC, Shepard JA, Shipley RT (1895). Computed tomography of localized pleural mesothelioma. AJR Am J Roentgenol.

[22] Ferretti GR, Chiles C, Cox JE, Choplin RH, Coulomb M (1997). Localized benign fibrous tumor of the pleura: MR appearance. J Comput Assist Tomogr.

[23] Podobnik J, Kocijancic I, Kovac V, Sersa I (2010). 3T MRI in evaluation of asbestos-related thoracic diseases - preliminary results. Radiol Oncol.

[24] Orki A, Kosar A, Akin A, Haclibrahimoglu G, Arman SB (2008). Solitary fibrous tumor of the pleura. Thorac Cardiovasc Surg.

[25] Gaint DT, Bokharl A, Shatt S, Dogra V (2011). Imaging features of solitary fibrous tumors. AJR Am J Roentgenol.

[26] Sung HS, Chang J-W, Kim J, Lee KS, Han J, Park S (2005). Solitary fibrous tumors of the pleura: surgical outcome and clinical cozrse. Ann Thorac Surg.

[27] Suter M, Gebhard S, Boumghard M, Peloponisions N, Genton CY (1998). Localized fibrous tumor of the pleura: 15 new cases and rewiev of the literature. Eur J Cardiothorac Surg.

[28] Crnjac A, Antonic J, Zorko A, Veingerl B (2001). Video-assisted thoracic surgery - a new possibility for the management of traumatic hemothorax. Wien Klin Wochenschr.

[29] Veronesi G, Spaggiari L, Mazzarol G, De Pas M, Leo F, Solli P (2000). Huge malignant localized fibrous tumor of the pleura. J Cardiovasc Surg.

[30] Park MS, Patel SR, Ludwig JA, Trent JC, Conrad CA, Lazar AJ (2011). Activity of temozolomide and bevacizumab in the treatment of locally advancet, recurrent and metastatic hemangiopericytoma and malignant solitary fibrous tumor. Cancer.

[31] De Perrot M (2000). Fibrous tumors of the pleura. Curr Treat Opt Oncol.

[32] Bicer M, Yaldiz S, Gursoy S, Ulgan M (1998). A case of giant benign localized fibrous tumor of the pleura. Eur J Cardiothorac Surg.

[33] Tublin ME, Tessler FN, Rifkin MD (1998). US case of the cay. Solitary fibrous tumor of the pleura (SFTP). Radiographic.

[34] Fan F, Hiu Z, Qinghai Z, Yupeng L (2014). Computed tomography manifestations of malignant solitary fibrous tumor of the pleura with distinct blood supply from celiac trunk. Eur J Cardiothorac Surg.

[35] Weynand B, Noel H, Goncette L, Noirhomme C, Colard P (1997). Solitary fibrous tumor of the pleura: a report of five cases diagnosed by transthoracic cuttig needle biopsy. Chest.

[36] Crnjac A (2004). The significance of thoracoscopic mechanical pleurodesis for the treatment of malignant pleural effusions. Wien Klin Wochenschr.

[37] Santos RS, Haddad R, Lima CE, Liu YL, Misztal M, Ferreira T (2005). Patterns of reccurence and long-term survival after curative resection of localized fibrous tunors of the pleura. Clin Lung Cancer.

